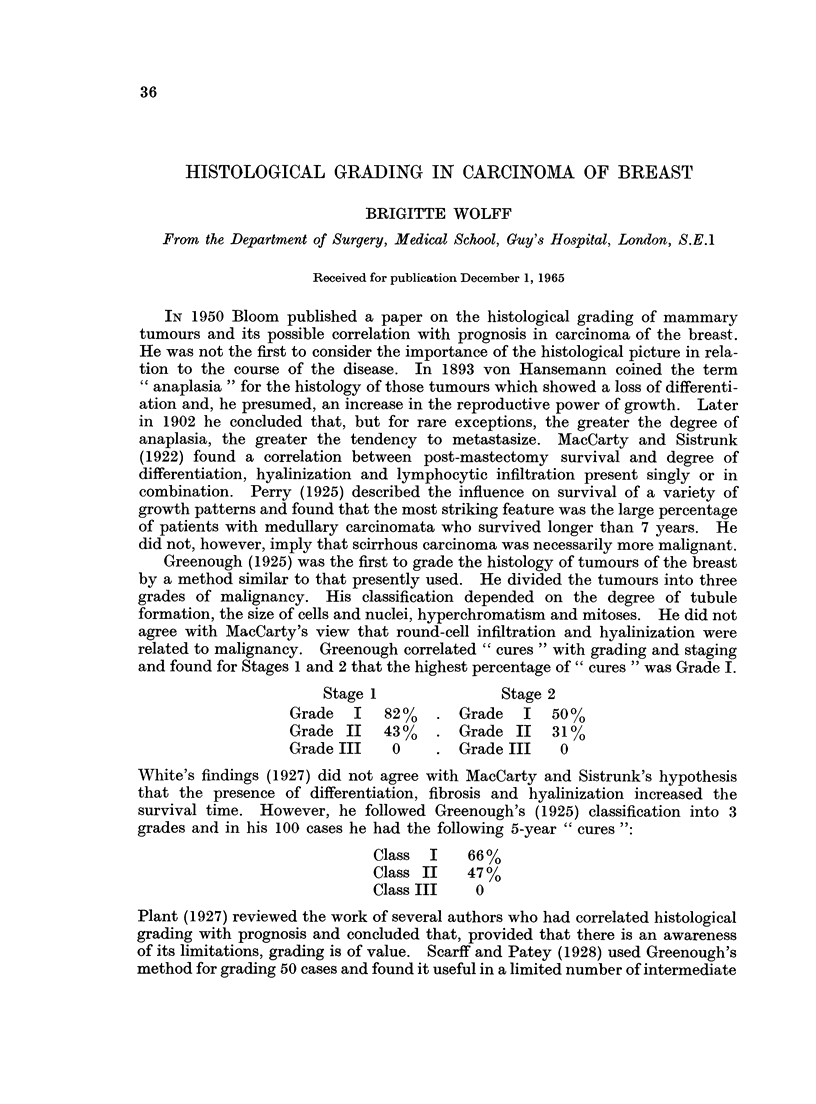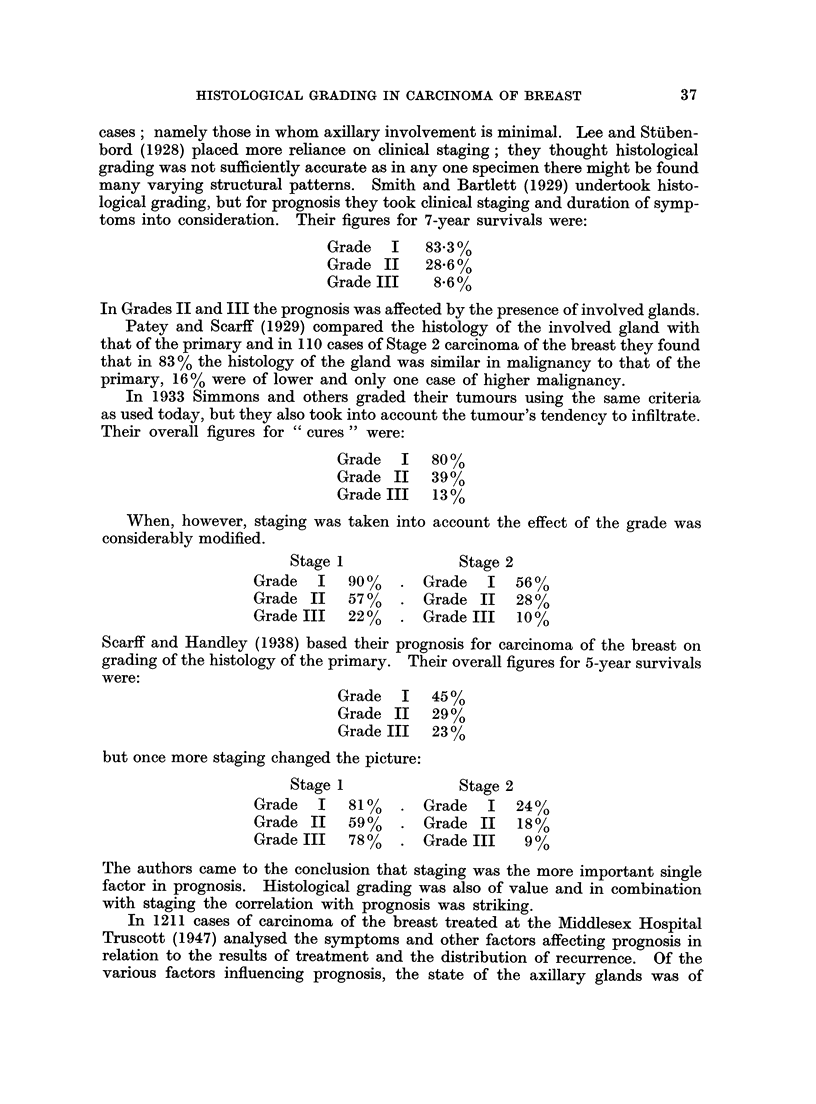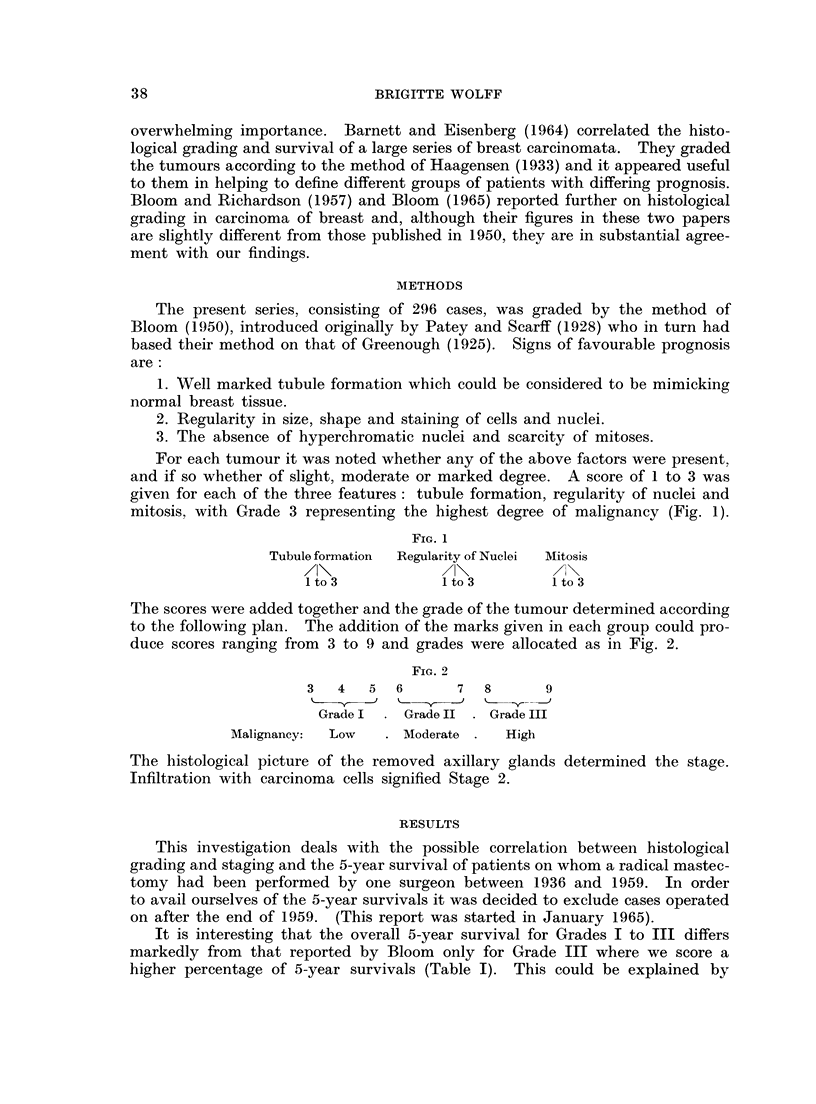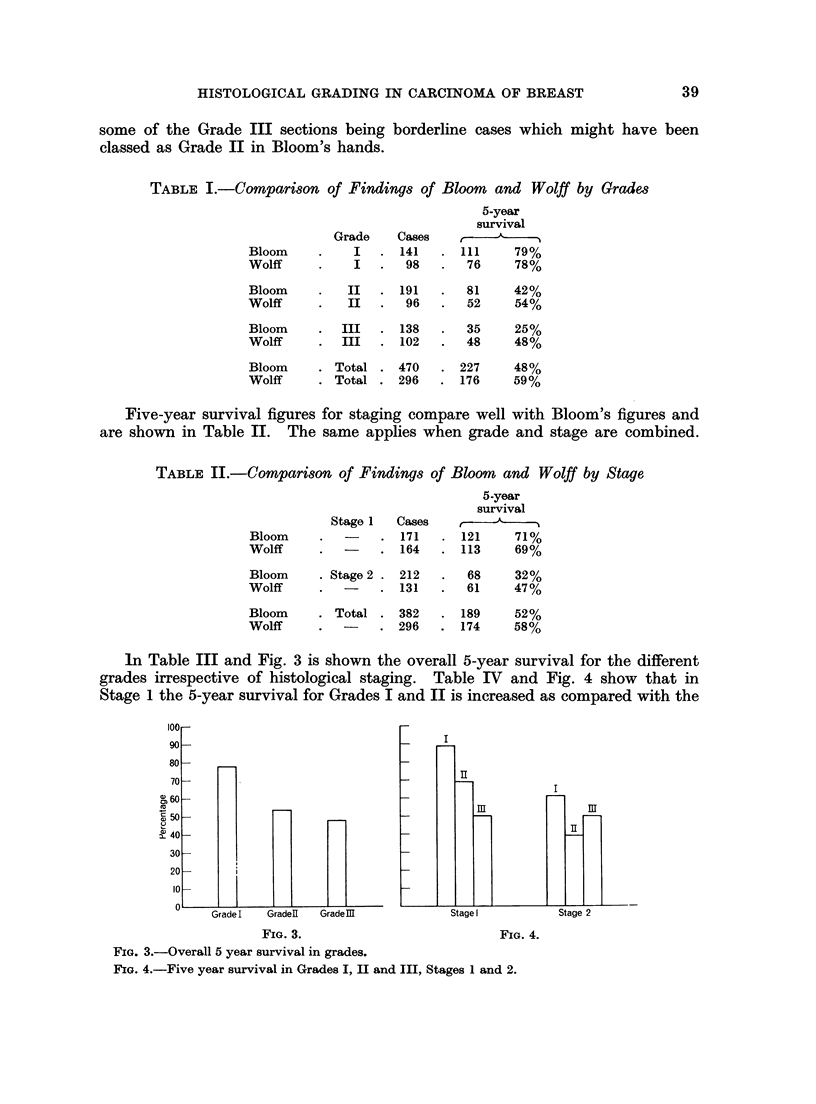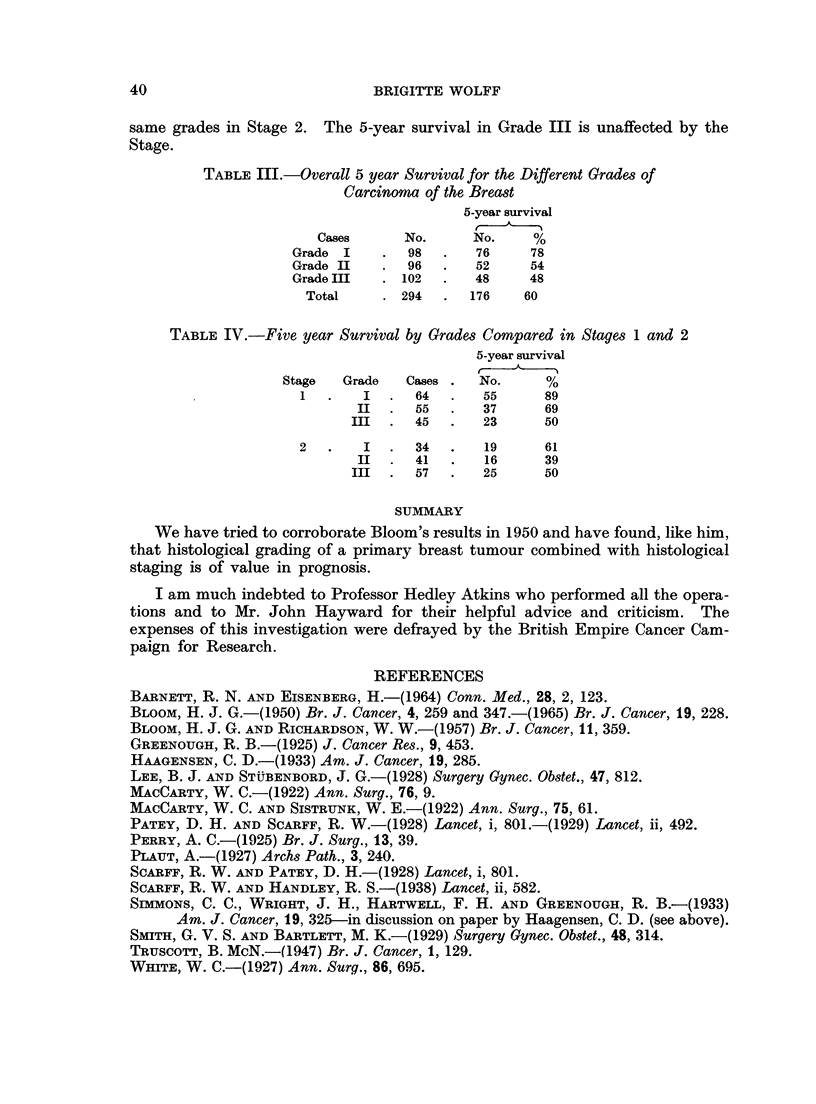# Histological grading in carcinoma of the breast.

**DOI:** 10.1038/bjc.1966.5

**Published:** 1966-03

**Authors:** B. Wolff


### HISTOLOGICAL GRADING IN CARCINOMA OF BREAST

BRIGITTE WOLFF

*From the Department of Surgery, Medical School, Guy's Hospital, London, S.E.*1

Received for publication December 1, 1965

IN 1950 Bloom published a paper on the histological grading of mammary tumours and its possible correlation with prognosis in carcinoma of the breast. He was not the first to consider the importance of the histological picture in relation to the course of the disease. In 1893 von Hansemann coined the term " anaplasia " for the histology of those tumours which showed a loss of differentiation and, he presumed, an increase in the reproductive power of growth. Later in 1902 he concluded that, but for rare exceptions, the greater the degree of anaplasia, the greater the tendency to metastasize. MacCarty and Sistrunk (1922) found a correlation between post-mastectomy survival and degree of differentiation, hyalinization and lymphocytic infiltration present singly or in combination. Perry (1925) described the influence on survival of a variety of growth patterns and found that the most striking feature was the large percentage of patients with medullary carcinomata who survived longer than 7 years. He did not, however, imply that scirrhous carcinoma was necessarily more malignant.

Greenough (1925) was the first to grade the histology of tumours of the breast by a method similar to that presently used. He divided the tumours into three grades of malignancy. His classification depended on the degree of tubule formation, the size of cells and nuclei, hyperchromatism and mitoses. He did not agree with MacCarty's view that round-cell infiltration and hyalinization were related to malignancy. Greenough correlated " cures " with grading and staging and found for Stages 1 and 2 that the highest percentage of " cures " was Grade I.

| Stage 1 | | | Stage 2 | |
|---|---|---|---|---|
| Grade I | 82% | . | Grade I | 50% |
| Grade II | 43% | . | Grade II | 31% |
| Grade III | 0 | . | Grade III | 0 |

White's findings (1927) did not agree with MacCarty and Sistrunk's hypothesis that the presence of differentiation, fibrosis and hyalinization increased the survival time. However, he followed Greenough's (1925) classification into 3 grades and in his 100 cases he had the following 5-year " cures ":

| | |
|---|---|
| Class I | 66% |
| Class II | 47% |
| Class III | 0 |

Plant (1927) reviewed the work of several authors who had correlated histological grading with prognosis and concluded that, provided that there is an awareness of its limitations, grading is of value. Scarff and Patey (1928) used Greenough's method for grading 50 cases and found it useful in a limited number of intermediate cases ; namely those in whom axillary involvement is minimal. Lee and Stübenbord (1928) placed more reliance on clinical staging ; they thought histological grading was not sufficiently accurate as in any one specimen there might be found many varying structural patterns. Smith and Bartlett (1929) undertook histological grading, but for prognosis they took clinical staging and duration of symptoms into consideration. Their figures for 7-year survivals were:

| | |
|---|---|
| Grade I | 83·3% |
| Grade II | 28·6% |
| Grade III | 8·6% |

In Grades II and III the prognosis was affected by the presence of involved glands.

Patey and Scarff (1929) compared the histology of the involved gland with that of the primary and in 110 cases of Stage 2 carcinoma of the breast they found that in 83% the histology of the gland was similar in malignancy to that of the primary, 16% were of lower and only one case of higher malignancy.

In 1933 Simmons and others graded their tumours using the same criteria as used today, but they also took into account the tumour's tendency to infiltrate. Their overall figures for " cures " were:

| | |
|---|---|
| Grade I | 80% |
| Grade II | 39% |
| Grade III | 13% |

When, however, staging was taken into account the effect of the grade was considerably modified.

| Stage 1 | | | Stage 2 | |
|---|---|---|---|---|
| Grade I | 90% | . | Grade I | 56% |
| Grade II | 57% | . | Grade II | 28% |
| Grade III | 22% | . | Grade III | 10% |

Scarff and Handley (1938) based their prognosis for carcinoma of the breast on grading of the histology of the primary. Their overall figures for 5-year survivals were:

| | |
|---|---|
| Grade I | 45% |
| Grade II | 29% |
| Grade III | 23% |

but once more staging changed the picture:

| Stage 1 | | | Stage 2 | |
|---|---|---|---|---|
| Grade I | 81% | . | Grade I | 24% |
| Grade II | 59% | . | Grade II | 18% |
| Grade III | 78% | . | Grade III | 9% |

The authors came to the conclusion that staging was the more important single factor in prognosis. Histological grading was also of value and in combination with staging the correlation with prognosis was striking.

In 1211 cases of carcinoma of the breast treated at the Middlesex Hospital Truscott (1947) analysed the symptoms and other factors affecting prognosis in relation to the results of treatment and the distribution of recurrence. Of the various factors influencing prognosis, the state of the axillary glands was of overwhelming importance. Barnett and Eisenberg (1964) correlated the histological grading and survival of a large series of breast carcinomata. They graded the tumours according to the method of Haagensen (1933) and it appeared useful to them in helping to define different groups of patients with differing prognosis. Bloom and Richardson (1957) and Bloom (1965) reported further on histological grading in carcinoma of breast and, although their figures in these two papers are slightly different from those published in 1950, they are in substantial agreement with our findings.

### METHODS

The present series, consisting of 296 cases, was graded by the method of Bloom (1950), introduced originally by Patey and Scarff (1928) who in turn had based their method on that of Greenough (1925). Signs of favourable prognosis are :

1. Well marked tubule formation which could be considered to be mimicking normal breast tissue.
2. Regularity in size, shape and staining of cells and nuclei.
3. The absence of hyperchromatic nuclei and scarcity of mitoses.

For each tumour it was noted whether any of the above factors were present, and if so whether of slight, moderate or marked degree. A score of 1 to 3 was given for each of the three features : tubule formation, regularity of nuclei and mitosis, with Grade 3 representing the highest degree of malignancy (Fig. 1).

FIG. 1

| Tubule formation | Regularity of Nuclei | Mitosis |
|---|---|---|
| 1 to 3 | 1 to 3 | 1 to 3 |

The scores were added together and the grade of the tumour determined according to the following plan. The addition of the marks given in each group could produce scores ranging from 3 to 9 and grades were allocated as in Fig. 2.

FIG. 2

| | 3 4 5 | 6 7 | 8 9 |
|---|---|---|---|
| | Grade I . | Grade II . | Grade III |
| Malignancy: | Low . | Moderate . | High |

The histological picture of the removed axillary glands determined the stage. Infiltration with carcinoma cells signified Stage 2.

### RESULTS

This investigation deals with the possible correlation between histological grading and staging and the 5-year survival of patients on whom a radical mastectomy had been performed by one surgeon between 1936 and 1959. In order to avail ourselves of the 5-year survivals it was decided to exclude cases operated on after the end of 1959. (This report was started in January 1965).

It is interesting that the overall 5-year survival for Grades I to III differs markedly from that reported by Bloom only for Grade III where we score a higher percentage of 5-year survivals (Table I). This could be explained by some of the Grade III sections being borderline cases which might have been classed as Grade II in Bloom's hands.

TABLE I.—*Comparison of Findings of Bloom and Wolff by Grades*

| | Grade | Cases | 5-year survival | |
|---|---|---|---|---|
| Bloom | I | 141 | 111 | 79% |
| Wolff | I | 98 | 76 | 78% |
| Bloom | II | 191 | 81 | 42% |
| Wolff | II | 96 | 52 | 54% |
| Bloom | III | 138 | 35 | 25% |
| Wolff | III | 102 | 48 | 48% |
| Bloom | Total | 470 | 227 | 48% |
| Wolff | Total | 296 | 176 | 59% |

Five-year survival figures for staging compare well with Bloom's figures and are shown in Table II. The same applies when grade and stage are combined.

TABLE II.—*Comparison of Findings of Bloom and Wolff by Stage*

| | Stage 1 | Cases | 5-year survival | |
|---|---|---|---|---|
| Bloom | — | 171 | 121 | 71% |
| Wolff | — | 164 | 113 | 69% |
| Bloom | Stage 2 | 212 | 68 | 32% |
| Wolff | — | 131 | 61 | 47% |
| Bloom | Total | 382 | 189 | 52% |
| Wolff | — | 296 | 174 | 58% |

In Table III and Fig. 3 is shown the overall 5-year survival for the different grades irrespective of histological staging. Table IV and Fig. 4 show that in Stage 1 the 5-year survival for Grades I and II is increased as compared with the

FIG. 3.

FIG. 4.

FIG. 3.—Overall 5 year survival in grades.

FIG. 4.—Five year survival in Grades I, II and III, Stages 1 and 2.

same grades in Stage 2. The 5-year survival in Grade III is unaffected by the Stage.

TABLE III.—*Overall 5 year Survival for the Different Grades of Carcinoma of the Breast*

| Cases | No. | 5-year survival No. | 5-year survival % |
|---|---|---|---|
| Grade I | 98 | 76 | 78 |
| Grade II | 96 | 52 | 54 |
| Grade III | 102 | 48 | 48 |
| Total | 294 | 176 | 60 |

TABLE IV.—*Five year Survival by Grades Compared in Stages* 1 *and* 2

| Stage | Grade | Cases | 5-year survival No. | 5-year survival % |
|---|---|---|---|---|
| 1 | I | 64 | 55 | 89 |
| | II | 55 | 37 | 69 |
| | III | 45 | 23 | 50 |
| 2 | I | 34 | 19 | 61 |
| | II | 41 | 16 | 39 |
| | III | 57 | 25 | 50 |

### SUMMARY

We have tried to corroborate Bloom's results in 1950 and have found, like him, that histological grading of a primary breast tumour combined with histological staging is of value in prognosis.

I am much indebted to Professor Hedley Atkins who performed all the operations and to Mr. John Hayward for their helpful advice and criticism. The expenses of this investigation were defrayed by the British Empire Cancer Campaign for Research.

### REFERENCES

BARNETT, R. N. AND EISENBERG, H.—(1964) *Conn. Med.*, **28**, 2, 123.
BLOOM, H. J. G.—(1950) *Br. J. Cancer*, **4**, 259 and 347.—(1965) *Br. J. Cancer*, **19**, 228.
BLOOM, H. J. G. AND RICHARDSON, W. W.—(1957) *Br. J. Cancer*, **11**, 359.
GREENOUGH, R. B.—(1925) *J. Cancer Res.*, **9**, 453.
HAAGENSEN, C. D.—(1933) *Am. J. Cancer*, **19**, 285.
LEE, B. J. AND STÜBENBORD, J. G.—(1928) *Surgery Gynec. Obstet.*, **47**, 812.
MACCARTY, W. C.—(1922) *Ann. Surg.*, **76**, 9.
MACCARTY, W. C. AND SISTRUNK, W. E.—(1922) *Ann. Surg.*, **75**, 61.
PATEY, D. H. AND SCARFF, R. W.—(1928) *Lancet*, i, 801.—(1929) *Lancet*, ii, 492.
PERRY, A. C.—(1925) *Br. J. Surg.*, **13**, 39.
PLAUT, A.—(1927) *Archs Path.*, **3**, 240.
SCARFF, R. W. AND PATEY, D. H.—(1928) *Lancet*, i, 801.
SCARFF, R. W. AND HANDLEY, R. S.—(1938) *Lancet*, ii, 582.
SIMMONS, C. C., WRIGHT, J. H., HARTWELL, F. H. AND GREENOUGH, R. B.—(1933) *Am. J. Cancer*, **19**, 325—in discussion on paper by Haagensen, C. D. (see above).
SMITH, G. V. S. AND BARTLETT, M. K.—(1929) *Surgery Gynec. Obstet.*, **48**, 314.
TRUSCOTT, B. MCN.—(1947) *Br. J. Cancer*, **1**, 129.
WHITE, W. C.—(1927) *Ann. Surg.*, **86**, 695.